# Supplementary material for: Chromatin profiling for everyone: FFPE-CUTAC for the theory and practice of modern molecular biology
Source: Front Epigenet Epigenom. 2026 Jan 26;3:1753710. doi: 10.3389/freae.2025.1753710 (PMC13639292; doi:10.3389/freae.2025.1753710)
Supplement: Supplementary file 1 [file Presentation1.pdf]

Checklist for FFPE-CUTAC experiments

Based on https://www.protocols.io/view/cutac-for-ffpes-14egn292zg5d/v4

1. **Deparaffinization**
   – Safeclear 2 in Coplin jar
   – EtOH series (100%, 50%, 10%) in Coplin jars
   – Xlink reversal in Coplin jar (85˚ 1hr)

2. **1˚ antibody binding**
   – 25 µL with saran wrap cover

3. **2˚ antibody binding**
   – rinse
   – 25 µL with saran wrap cover

4. **pAGTn5 binding**
   – rinse
   – 25 µL pAGTn5 dilution
   – Coplin jar to rinse

5. **Tagmentation**
   – in Coplin jar (55˚ 1hr)

6. **Fragment release**
   – scrape into tubes
   – SDS/protease K incubation in PCR machine
   – quench with triton-X100

7. **PCR**
   – set up reactions on ice
   – run PCR program

8. **Ampure (SPRI) cleanup**
   – add SPRI beads
   – use magnets, wash with 80% EtOH
   – elute with 10 mM Tris pH8

9. **tapestation**
   – run!
